# A Local Agreement Filtering Algorithm for Transmission EM Reconstructions

**DOI:** 10.1016/j.jsb.2018.11.011

**Published:** 2019-01-01

**Authors:** Kailash Ramlaul, Colin M. Palmer, Christopher H.S. Aylett

**Affiliations:** a Section for Structural Biology, Department of Medicine, Imperial College Road, South Kensington, London SW7 2BB, United Kingdom; b Scientific Computing Department, Science and Technology Facilities Council, Research Complex at Harwell, Didcot OX11 0FA, United Kingdom

**Keywords:** 2D/3D, 2/3-Dimensional, Cryo-EM, Electron Cryo-Microscopy, CDF, Cumulative distribution function, EM, Electron microscopy, FSC, Fourier shell correlation, LAFTER, Local Agreement Filter for Transmission EM Reconstructions, RMSD, Root mean squared deviation, SNR, Signal to noise ratio, xFSC, Cross-FSC, C_ref_, Cryo-EM, Local resolution, Noise suppression, Real-space filter

## Abstract

We present LAFTER, an algorithm for de-noising single particle reconstructions from cryo-EM.

Single particle analysis entails the reconstruction of high-resolution volumes from tens of thousands of particle images with low individual signal-to-noise. Imperfections in this process result in substantial variations in the local signal-to-noise ratio within the resulting reconstruction, complicating the interpretation of molecular structure. An effective local de-noising filter could therefore improve interpretability and maximise the amount of useful information obtained from cryo-EM maps.

LAFTER is a local de-noising algorithm based on a pair of serial real-space filters. It compares independent half-set reconstructions to identify and retain shared features that have power greater than the noise. It is capable of recovering features across a wide range of signal-to-noise ratios, and we demonstrate recovery of the strongest features at Fourier shell correlation (FSC) values as low as 0.144 over a 256^3^-voxel cube. A fast and computationally efficient implementation of LAFTER is freely available.

We also propose a new way to evaluate the effectiveness of real-space filters for noise suppression, based on the correspondence between two FSC curves: 1) the FSC between the filtered and unfiltered volumes, and 2) C_ref_, the FSC between the unfiltered volume and a hypothetical noiseless volume, which can readily be estimated from the FSC between two half-set reconstructions.

## Highlights

• We propose an algorithm, LAFTER, that recovers features with more signal than noise from half maps.
• LAFTER is shown to recover features over a wide range of FSCs and local signal-to-noise ratios.
• We suggest effective local noise suppression be evaluated by comparing the filter-sum xFSC to C_ref_.

## Graphical abstract

**Figure:**
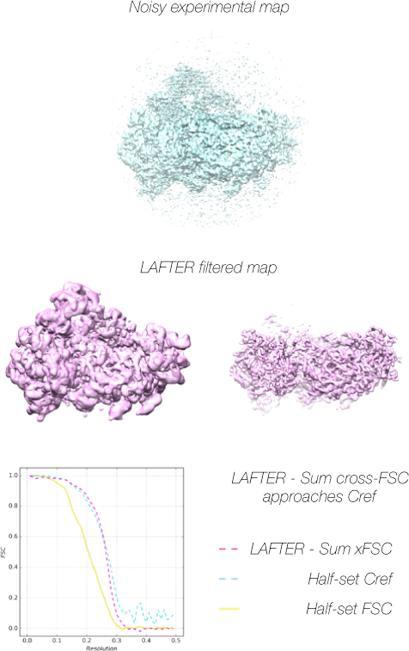

## 1 Introduction

### 1.1 Interpretability of single particle density maps in Fourier-space

Single particle analysis entails the *in silico* reconstruction of high-resolution volumes from (typically) tens of thousands of transmission electron particle images of vitrified specimens with low individual signal-to-noise ratios (*SNRs*) (Frank, 1975, Adrian et al., 1984, Henderson et al., 1990). Whereas in crystallography, helical diffraction or nuclear-magnetic resonance spectroscopy, the experimenter has an immediate read-out of the quality of their data (the reflections, layer-lines and peaks respectively), single particle analysis is denied an accurate estimate of the eventual resolution or SNR without extensive data processing. Although the two-dimensional (2D) information limit may now be estimated quickly (Rohou and Grigorieff, 2015, Zhang, 2016), three-dimensional (3D) analysis is limited by the incredibly low SNR recorded for individual particles before their signal fades due to radiation damage, and the fact that the 3D Fourier space must be reconstructed from heterogeneous, preferentially distributed 2D projections (Saxton and Frank, 1977, Henderson, 1995). This is a real issue, as it is impossible to interpret reconstructed densities without knowing what features can be relied upon.

Scientists have struggled with this problem since the field began (Liao and Frank, 2010). Initial efforts focused on a resolution limit in Fourier space (Frank et al., 1981, Kessel et al., 1985, van Heel, 1987, Harauz and van Heel, 1986, Penczek, 2002, Unser et al., 2005, Sousa and Grigorieff, 2007), and today this is commonly measured using the Fourier shell correlation (*FSC*) (Harauz and van Heel, 1986). The FSC is frequently calculated between independently reconstructed half-sets, in order to prevent the correlation of one half-density with noise from the other during the reconstruction process (Grigorieff, 2000, Henderson et al., 2012).

It is generally acknowledged that the SNR in Fourier space varies according to the distribution of the particle projections; the frequency shells are therefore non-uniform (e.g. Tan et al., 2017). However, the field has converged towards the use of a set of criteria for the resolution at which a reconstruction should be low-pass filtered. Some of these take into account the effects of applying symmetry or voxel count, such as the ½-bit FSC criterion (van Heel and Schatz, 2005), or the masking of volumes (Chen et al., 2013), whereas others do not. Most criteria lie in the same vicinity for an unmasked map, without symmetry averaging, at high resolution. This convergence is based on the calculation that an FSC of 0.143 would correspond to a theoretical cross-FSC (*xFSC*), or figure of merit, C_ref_ of 0.5 between the summed experimental half maps and a “noiseless” map (Rosenthal and Henderson, 2003). A figure of merit of 0.5 was already a standard for dataset phasing in crystallography, and using a similar interpretability measure for cryo-EM data is desirable.

### 1.2 Local resolution and density map interpretability in real-space

The interpretability of single particle reconstructions also varies in real-space. This occurs because the density is reconstructed from many images of different particles. Regions that vary between the particles will exhibit a mixture of their respective signals. Several sources are known to contribute to this phenomenon, principally partial occupancy (Belnap et al., 2003), and conformational variability (Ishikawa et al., 2004, Leschziner and Nogales, 2007). Affected reconstructions have a lower SNR in the varying regions. The variations between particles are often relatively localised in their effects and therefore this phenomenon has been dubbed “local resolution”, and handled accordingly.

The first attempts to diagnose local resolution rigorously began with the work of Cardone et al. (2013), who demonstrated that local variations could be identified similarly to global resolution. They convoluted a window function with the reconstruction and calculated the half-set FSC locally at each position. Due to an effect of the properties of the window on the outcome, their method was less widely adopted than it deserved. The most widely used algorithm for local resolution determination currently is that of Kucukelbir et al. (2014). They convoluted the reconstruction with a series of small kernels forming a complete basis for the extraction of the local waveform at a given resolution. This increased the resolution and reproducibility with which local resolution could be determined. A further recent step forward has been the demonstration of the direct extraction of amplitudes for SNR estimation using the monogenic signal by Vilas et al. (2018).

### 1.3 Current real-space filters for local noise suppression

Without a way of representing volumes according to their interpretability, local resolution estimates would be of limited use. The initial approach to solving the issues posed by local resolution was to isolate regions from the reconstruction, and then to mask and filter each (Gao et al., 2004). The development of programs to diagnose local resolution allowed these methods to be reversed, filtering the volume according to the local resolution assigned to each voxel. BLOCFILT (Cardone et al., 2013) was the first algorithm to do this, while the local filter provided by RELION (Chen et al., 2013) performs a similar process. Few real-space filters are currently available; the MONORES algorithm (Vilas et al., 2018) provides one that considers only the minimal vicinity of the voxel for each frequency, rather than incorporating noise from the solvent as windowing methods do.

### 1.4 Local resolution filtering and signal to noise filtering

The philosophy underlying previous local filtering approaches has very closely mirrored the approach taken for global noise. The signal over a substantial window of the map in each case is truncated in the Fourier domain, or an extension of it in the case of MONORES, at a point at which the signal is considered to have fallen below an acceptable level. Typically noise suppression has not been an aim in and of itself. Instead, interpretation has been seen from the perspective of the “usable” resolution.

We approach the problem of signal-to-noise ratio within the experimental map from a different perspective. We have set out to provide a filter to produce a locally noise-suppressed map in real space, using Fourier space as a tool for deconvolution of the noise from the signal where appropriate. We seek to both weight and truncate the experimental map in order to ensure as much representation of signal and as little representation of noise as possible, without consideration of resolution in and of itself.

### 1.5 Cause for Local Agreement Filtering of Transmission EM Reconstructions (LAFTER)

A consideration of the real-space filtering problem reveals that the aims of local resolution diagnosis are at odds with those of a filter to provide a noise-suppressed map. The principal concerns of diagnostic tools must be: to step finely through the resolution range to maximise resolution assignment accuracy; to be locally consistent to allow interpretation of the local-resolution map; and to provide a binary, “resolved/unresolved” statement of significance against a certain p-value for each voxel at each resolution. Only the consistency requirement applies to filtering, while a binary assignment is damaging as it fails to reflect the reality of the SNR continuum within all reconstructions.

An algorithm for noise minimisation must step through the lowest and highest resolution shells most finely, as the variation due to the signal is highest at low resolution, whereas the large variation due to noise must be suppressed effectively at high resolution. There is no requirement for fine slicing when the SNR is very high. It can also take into account the known increase in noise with higher resolution, and the fact that, in a macromolecule, signal from the same structure will be present at all length scales currently accessible to cryo-EM. It may function at any resolution below the resolution limit of the map without compromising the results, not being limited to twice the reciprocal separation in question, thus yielding relatively crisp edges to avoid blurring the density into the solvent. Finally it is desirable that the filter should weight the map appropriately, rather than truncating at a particular frequency.

### 1.6 Evaluating the LAFTER denoising algorithm and a general means by which to evaluate the effectiveness of local noise suppression

We present a real-space filtering algorithm, a Local Agreement Filter for Transmission EM Reconstructions (LAFTER), which filters maps according to an estimate of the noise distribution and thereby facilitates interpretation. It is important to note that LAFTER is not a “local resolution” filter *per se*, as local resolution is not explicitly evaluated; instead it focuses on the SNR. The SNR is higher for regions of stronger density, and therefore LAFTER frequently allows these regions to be observed through underlying noise even if the local resolution is poor. This phenomenon is often observed in the case of phosphates in nucleotide residues. A disadvantage of this SNR-centric approach is that regions of the molecule with lower SNR must be observed at lower contours.

We have tested LAFTER both on synthetic data and on experimental reconstructions, and detail the results here. In order to evaluate the success or failure of LAFTER in suppressing the noise between half-maps, we note that, as noise suppression is achieved, the xFSC between the filtered volume and the unfiltered volume should approach C_ref_, the estimated xFSC between the unfiltered volume and a theoretical noiseless volume. We apply this correspondence as a measure of successful noise suppression by LAFTER, and show that the xFSC for LAFTER output maps indeed approaches C_ref_. While unsuitable as a target for optimisation, we suggest that a comparison between these curves represents a sensible global measure by which to evaluate the efficient suppression of noise by local filters in general.

## 2 Methods

### 2.1 The LAFTER algorithm

#### 2.1.1 Summary

**Fig. 1:**
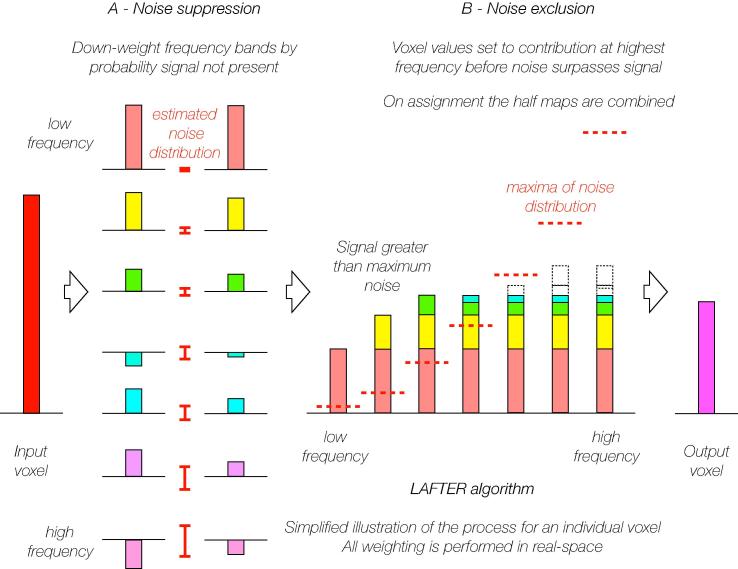
The LAFTER algorithm. The application of the LAFTER algorithm is illustrated graphically for an individual voxel. Magnitude is indicated by the height (or depth) of the bar, and the relevant properties of the noise within the dataset as a whole are indicated by red lines.

The intention of LAFTER is to suppress noise to the point at which every voxel within the final map has an SNR greater than 1.0. LAFTER operates in both real and Fourier space. It consists of two separate sequential filters. The first isolates and weights frequency bands independently according to the probability they represent noise (Fig. 1A), to suppress the noise in the map. The second filter excludes higher frequency noise where it could be stronger than the signal (Fig. 1B). The resulting map should have high SNRs throughout.

#### 2.1.2 Assumptions

Our algorithm makes three explicit assumptions. First, we assume that the agreement and disagreement between half-maps represent signal and noise respectively. This assumption is also inherent to the FSC calculation, and is required to estimate the signal and noise distributions, however it is frequently violated to some extent due to over-refinement, an unavoidable artefact of certain refinement practices. Second, we assume that noise between half-maps is Gaussian at each resolution, and well distributed within the refined region. This is necessary as we estimate the power of the noise at each resolution, and this assumption has proven safe in all experimental cases tested, providing that only the region that was refined is considered. Finally, we assume that features have structure that varies smoothly across successive resolution bands. This assumption is necessary as LAFTER operates on isolated (band-pass filtered) resolution bands in real-space, but we consider it safe for macromolecular samples as biological structures can be considered smooth and exhibit structure at all scales up to the current resolution limit of the technique.

#### 2.1.3 Steps in the algorithm

LAFTER performs two successive filtering operations on the half-volumes (Fig. 1). In the first, resolution shells are isolated from the two half volumes by band-pass filtering. The half volumes are transformed into Fourier space, and for each resolution shell, the Fourier coefficients are weighted using an eighth-order Butterworth band-pass filter (Butterworth, 1930):

$$F_{\mathrm{out}}\left(r\right)=F_{\mathrm{in}}\left(r\right)∙\left(\sqrt{\frac{1}{1+{\left(\frac{r}{h}\right)}^{16}}}-\sqrt{\frac{1}{1+{\left(\frac{r}{l}\right)}^{16}}}\right)$$

In this equation, $F_{\mathrm{in}}\left(r\right)$ and $F_{\mathrm{out}}\left(r\right)$ represent the complex Fourier coefficients in the original transform and the band-passed output respectively, at radius r from the origin; h and l are the high and low cutoff frequencies for the band-pass filter. This particular filter is used because it produces minimal ringing artefacts and conveniently sums to unity across successive resolution bands, meaning no further scaling is necessary when the filtered volumes are later re-combined.

Each successive resolution shell is incorporated using an adaptive step size proportional to the current resolution and the current mean signal probability (see below). This minimises the step size both at low resolution, where the signal varies strongly, and near the resolution-limit, as the noise eclipses the signal. The larger the step the algorithm takes, the greater its chance of estimating the noise incorrectly, or of introducing discontinuities into the signal. The incorporation of new resolution shells terminates when the FSC between half-maps reaches either a threshold of 0.143, or a user-provided threshold value.

For each resolution shell, the two half volumes are transformed to real space after the band pass filter has been applied. The total power at this resolution, T, and the power of the noise, N, are calculated from the sums and differences of the voxel values respectively:

$$T=\sum_{\mathrm{xyz}}{\left(v_{1,xyz}+v_{2,xyz}\right)}^{2}$$

$$N=\sum_{\mathrm{xyz}}{\left(v_{1,xyz}-v_{2,xyz}\right)}^{2}$$

In these equations, $v_{1,xyz}$ and $v_{2,xyz}$ are the values of the voxels from the two half-volumes at position xyz, and the sum is over all voxel positions within the region of the map that was considered during reconstruction.

The standard deviation of the noise is calculated as $\sigma_{N}=\sqrt{N/n}$, where n is the number of voxels within the region considered, and the proportional contributions of the noise and the signal to the total power are calculated as follows:

$$P_{N}=\frac{N}{T}$$

$$P_{S}=1-P_{N}$$

Each voxel in each of the band-passed half volumes is then weighted using an estimate of the probability that it corresponds to signal. This is calculated using erf, the error function for the normal distribution:

$$\mathrm{erf}\left(x\right):=\frac{2}{\sqrt{\pi }}\int_{0}^{x}e^{{-t}^{2}}dt$$

First, erf is used to calculate the probability of measuring a value at least as great in magnitude as the voxel value, assuming it is sampled from the noise distribution:

$$P\left(noise\right)=1-erf\left(\frac{\left|v_{1,xyz}+v_{2,xyz}\right|}{\sqrt{2}∙\sigma_{N}}\right)$$

This is then used to estimate the overall probability that the voxel corresponds to signal according to the following formula:

$$P\left(signal\right)=\frac{P_{S}∙\left(1-P\left(noise\right)\right)}{P_{N}∙P\left(noise\right)+P_{S}∙\left(1-P\left(noise\right)\right)}$$

This calculation can be seen as a best attempt at prior adjustment of the signal probability for this voxel, using the overall signal contribution to the power at the given resolution as a prior. Rigorous Bayesian adjustment would require estimation of the probability distribution corresponding to the signal, which is too computationally expensive to estimate for routine use, and does not appear to greatly affect the output except at very low SNRs. A modified version of *LAFTER* that estimates the signal distribution through Maximum Likelihood is available on request from the authors. We note that it is not fast.

The voxel values in real space are weighted using this probability estimate (Fig. 1A), and normalised (to make them comparable for the second filter) by the resolution shell width and the root mean squared value of the total power at that resolution:

$$v_{\mathrm{out},xyz}=v_{\mathrm{in},xyz}∙\frac{P(signal)∙(h-l)}{\sqrt{T/n}}$$

This multiplication by the probability of significance has the benefit of suppressing noise considerably, without the substantial computation that is required to develop a statistical model of the signal, and can be thought of as “adaptive masking” of regions indistinguishable from noise.

Finally, the estimated probabilities for all voxels within the region of interest are used to calculate the mean signal probability for this resolution, which is used in the calculation of the next resolution step size (see above).

After all resolution shells have been processed, the series of band-passed, noise-weighted maps for each half volume is then summed in real space, combining the isolated resolutions to yield a pair of noise-suppressed half volumes.

In the second filtering step (Fig. 1B) the noise-suppressed half volumes from the first filter are transformed into the Fourier domain, and then each low pass filtered at every resolution that was considered in the previous step. Low pass filtering is performed similarly to the band pass filtering described above, using an eighth-order Butterworth response (Butterworth, 1930):

$$F_{\mathrm{out}}\left(r\right)=F_{\mathrm{in}}\left(r\right)∙\sqrt{\frac{1}{1+{\left(\frac{r}{h}\right)}^{16}}}$$

Each pair of low pass-filtered half-maps is transformed back into real space, and a summed volume is calculated. The noise maximum is found as the greatest difference between corresponding voxels in the half volumes, for all voxel coordinates xyz within the region considered:

$${\mathrm{noise}}_{\max}=max\left(\left|v_{1,xyz}-v_{2,xyz}\right|\right)$$

Starting at the highest resolution considered, each voxel in the summed volume is tested. If its value is greater than the maximum noise at the current resolution, then that value is assigned to the corresponding voxel in the final output volume. If its value is lower than the noise maximum, the corresponding voxel in the output volume is left un-assigned, and will be re-considered at the next (lower) resolution. Voxels that have already been assigned at higher resolution are excluded from consideration at lower resolutions, so the overall effect is that each voxel in the final output is assigned to its value at the highest resolution where its signal is greater than the maximum noise (Fig. 1B).

After the last, lowest-resolution pair of half-volumes has been processed, the output density map is slightly softened to remove hard edges, by setting any remaining zero-valued voxels to an average of their six nearest neighbours. (This is done eight times to ensure that the map density spreads smoothly into any un-assigned areas.) Finally, the volume is explicitly low-pass filtered at the highest resolution that was considered during the noise suppression process.

#### 2.1.4 Important points

LAFTER is intended to recover the signal corresponding to the agreement between two independently refined half-sets. It uses the noise distribution between half-sets, from which the FSC and therefore C_ref_ is calculated, and therefore requires the independent, unfiltered half-maps for agreement estimation, and a map or mask from 0 to 1, where 1 indicates the voxel was refined, specifying the region used in the refinement process, to identify those regions within which the noise distribution can safely be estimated. Over-fitting or over-refinement is a major issue and will typically invalidate the results, as the over-refined noise will be retained in the filtered map. We would encourage users to rethink their refinement strategy in such cases. Similarly, running LAFTER with an incorrect region of the map specified by the mask will also result in an incorrect result.

### 2.2 Implementation and availability

We have produced a reference implementation of LAFTER as a performance-optimised C program using FFTW for Fourier transformation (Frigo and Johnson, 2005) to maximise speed and portability. It performs acceptably in terms of speed and computing requirements in comparison to other local filters, typically processing a 256^3^ voxel volume on a 4-CPU 2.7 GHz x86-64 laptop in one and a half minutes. To simplify the use of LAFTER for macromolecular interpretation, we output an MRC format volume (Cheng et al., 2015) that is up-sampled to give a smooth map suitable for use with model building tools. Source code for the LAFTER reference implementation is available from the Imperial College Section for Structural Biology GitHub (github.com/StructuralBiology-ICLMedicine) under the GPL open source licence. It operates on MRC format density files in MRC mode 2 (C float or FORTRAN real). It is provided as a C-2000 program requiring the C standard library and FFTW3, and can be compiled for any POSIX-compatible operating system. LAFTER will also be made available in pre-compiled binary format for both Linux and Mac OS X as part of the CCP-EM suite (Burnley et al., 2017).

### 2.3 Synthetic and experimental datasets

Simulated half maps were generated by adding noise to noiseless densities, and simulated full reconstructions were generated by summing the two half maps. We used synthetic densities (3D models of Tux, Gnu and Mandelbulb – kindly provided by thingiverse users me2space, luigismith and aeron203 respectively) to provide a fully controllable benchmark with an immediately recognisable, strong signal that cannot be confused with an experimental volume even without close inspection. Gaussian noise of the necessary power to yield the desired half-set FSC (van Heel and Schatz, 2005) was generated in two dimensions (real and imaginary units) using the SciPy (Jones et al., 2001) function scipy.stats.multivariate_normal, with zero mean, zero covariance and the stated power in relation to the known (Fourier-space blurred) signal, and added directly to the Fourier transform of the noiseless maps using the same framework. When the FSC was varied, the noiseless map was low-pass filtered at a resolution of 4.0 voxels, while when the resolution was being varied the FSC was set at 0.5. Results for a series of macromolecular synthetic densities (based on the proteasome from PDBID 6BDF) are included in the supplement to show that they behave similarly.

Regions of lower local resolution typically have low SNR and therefore lower FSCs than the better-ordered regions of the density. LAFTER is intended to aid interpretation of regions of low local resolution, or weak SNR in general, with correspondingly low FSCs. If the global FSC varies before the resolution cut-off, any global filtering effect can improve the map. Because the LAFTER algorithm will have a coincidental global filtering effect, we used a low, flat FSC to ensure that any improvement in the map represents the effects of local filtering only. This excludes the interpretation that noise suppression might be a resolution shell, global filtering, effect.

We also show the results of LAFTER application to five experimental datasets corresponding to EMDB entries (EMD-3048, EMD-6721, EMD-3954, EMD-6287 and EMD-3460). These density maps were chosen firstly because of the availability of independently refined half-set density maps and models, and secondly in order to cover a wide range of local and global resolutions (∼2.5 to ∼25 Å) and of molecular structures (DNA, RNA and protein).

### 2.4 Calculation of C_ref_ and comparison to LAFTER-Sum xFSC

In order to evaluate the efficiency of noise suppression by LAFTER, we used a comparison between: 1) the FSC between the LAFTER-filtered and unfiltered volumes, and 2) the statistic C_ref_. The value of C_ref_ was calculated from the FSC between unfiltered half sets according to the equation defined by Rosenthal and Henderson:

$$C_{\mathrm{ref}}=\sqrt{\frac{2∙FSC}{1+FSC}}$$

C_ref_ provides an independent, widely accepted, estimator of the xFSC of the unfiltered volume with a (usually hypothetical) noiseless volume. For the synthetic maps we generated, we have the advantage of being able to calculate the true FSC between the original noiseless volume and the noisy volume derived from it. The C_ref_ estimate calculated from these maps agrees very well with the true map xFSC (RMSD below 0.01 for resolutions up to the cut-off), as is predicted by theory.

The xFSC between a locally filtered map and the unfiltered summed half-maps (*Filter-Sum xFSC*) reveals the level of residual noise at each resolution. Any global filter must necessarily yield a Filter-Sum xFSC of 1, as at each resolution the Fourier components in each Fourier shell will be scaled versions of one another. Any local filter must necessarily yield a Filter-Sum xFSC below 1, as the Fourier components in each Fourier shell will no longer be scaled equivalents, one density having been scaled in real-space without convolution. The Filter-Sum xFSC drops from 1.0, in the case of a global filter, to reach C_ref_ as the residual noise level is decreased to zero (Supplementary Fig. 1). In experimental cases, perfect deconvolution of the signal from the noise is of course impossible: there will always be some suppression of both signal and noise, however the Filter-Sum xFSC to C_ref_ comparison remains a clear indicator of the level of noise suppression (Supplementary Fig. 1).

We propose that the residual between C_ref_ and the Filter-Sum xFSC provides a useful measure by which to judge the success of a local filter in achieving noise suppression in the output map. The logic is as follows: a half-set sum to filtered volume xFSC higher than C_ref_ must indicate the retention of noise, as the signal alone could only yield a Filter-Sum xFSC equalling C_ref_, whereas a Filter-Sum xFSC below C_ref_ must indicate the loss of at least some of the useable signal available in the original data, as the Filter-Sum xFSC with a noiseless volume should yield C_ref_. A local filter suppressing noise optimally would be expected to yield maps with a Filter-Sum xFSC approaching Cref, as it must balance as evenly as possible the loss of usable signal with the retention of problematic noise.

All experimental FSCs were calculated with masked densities to maximise their effective comparison to the LAFTER output. This C_ref_ control is performed by the LAFTER reference implementation and reported to stdout, along with the corresponding RMSD between the curves, to provide the user with a measure of the effectiveness of noise suppression.

## 3 Results

### 3.1 LAFTER recovered input features from synthetic data at a range of resolutions

**Fig. 2:**
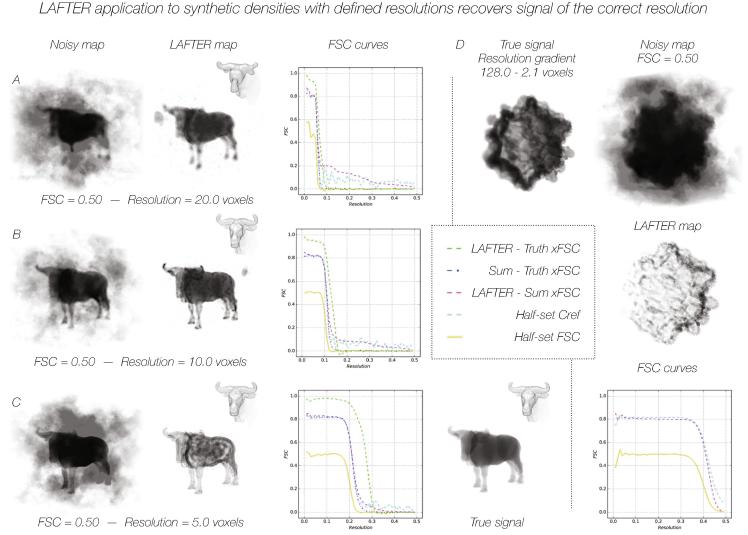
LAFTER application to synthetic densities with defined resolutions recovers signal of the correct resolution. LAFTER output for synthetic input truncated at resolutions of 20 voxels (A), 10 voxels (B) and 5 voxels (C) and over a 128.0–2.1 voxel resolution gradient (D). All synthetic input was constructed with a flat FSC of 0.5, and the same level of noise with respect to the noiseless structure maintained after the resolution cut-off. In each case the initial noisy and filtered maps are shown in grey with a linear transparency gradient over the density. The densities are shown as transparent “solids” as the signal in the half volumes is often otherwise indiscernible, with surface features inset. Curves for the half-set FSC, C_ref_ and xFSCs between the filtered, true and unfiltered maps in each case are shown adjacent as described in the key.

LAFTER was trialled against four synthetic datasets with explicitly generated resolution limits and smoothed, but approximately constant, SNR to ensure that the algorithm output exhibited the expected behaviour. To prevent confusion and ease interpretation, non-macromolecular maps were used (macromolecular output is shown in Supp. Fig. 2A–F). The algorithm was modified to prevent termination until the Nyquist limit was reached, in order to evaluate the efficacy of noise suppression at very low SNR, well above the resolution of the low-pass filter that was applied to the signal. The signal of the noiseless, softened, density maps was explicitly truncated at three different resolutions: 20.0, 10.0 and 5.0 voxels (Fig. 2A–C), and along a gradient from 128.0 to 2.1 voxels (Fig. 2D), before combination with Gaussian noise at an FSC of 0.5. In each case the resolution of the resulting LAFTER output volume increased along with the resolution of the signal exactly as expected. Some signal is lost completely, excluded by the algorithm, at lower resolution due to the signal falling below the noise (Fig. 2D).

### 3.2 LAFTER recovered input features from synthetic data at low SNR

**Fig. 3:**
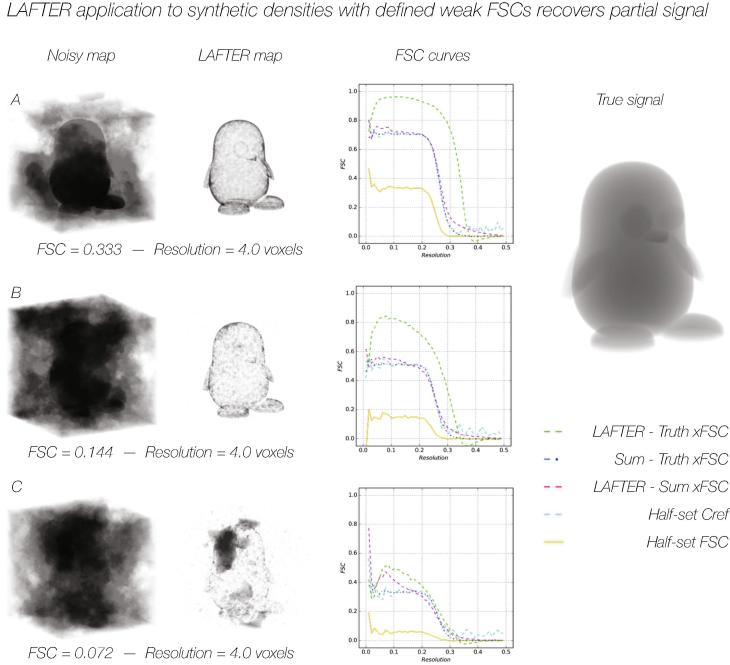
LAFTER application to synthetic densities with defined weak FSCs recovers partial signal. LAFTER output for synthetic input truncated at a resolution of 4 voxels and with FSCs of 0.333 (A), 0.144 (B) and 0.072 (C). All synthetic input was constructed with the same level of noise with respect to the noiseless structure throughout. In each case the initial noisy and filtered maps are shown in grey with a linear transparency gradient over the density. The densities are shown as transparent “solids” as the signal in the half volumes is often otherwise indiscernible. Curves for the half-set FSC, C_ref_ and xFSCs between the filtered, true and summed densities in each case are shown adjacent as described in the key.

LAFTER was trialled against further synthetic datasets with low SNR. This was an extreme case with sharp signal and massive noise, but was intended to explore the robustness of feature recovery without any effect of whole resolution shell weighting. The FSC for a map truncated at a resolution of 4.0 voxels was explicitly decreased from 0.333, to 0.144 and 0.072 (Fig. 3A–C). The quality of the recovered signal falls off at low SNR as expected, however LAFTER output has a higher xFSC with the noiseless input volume than the summed half maps down to a half-set FSC of 0.144, demonstrating the power of LAFTER.

### 3.3 The output of LAFTER gives a Filter-Sum xFSC approximating C_ref_ and a higher xFSC against the true volumes

The expected FSC between the summed noisy volume and an idealised noiseless volume should be approximated by the calculated value of C_ref_ from the FSC between the two noisy half-sets. This is the case for the synthetic data we generated (Fig. 2A-C; Fig. 3A-C). The expectation would be that the FSC between an effectively noise-suppressed volume and the summed noisy half sets should yield a similar curve to C_ref_, and the FSC between the filtered volume and the noiseless synthetic volume should be substantially higher (ideally approaching unity, however this must remain beyond the scope of any algorithm in regions of low SNR as there is insufficient information retained). When these curves are evaluated, this is indeed generally the case, although as the SNR and FSC decrease to low levels LAFTER performs poorly, as would be expected (Fig. 2A-C; Fig. 3A-C). Although a systematic investigation has not been performed, generally LAFTER performs noticeably better than other local filters on densities with low FSCs (Supp. Fig. 3A-C).

**Fig. 4:**
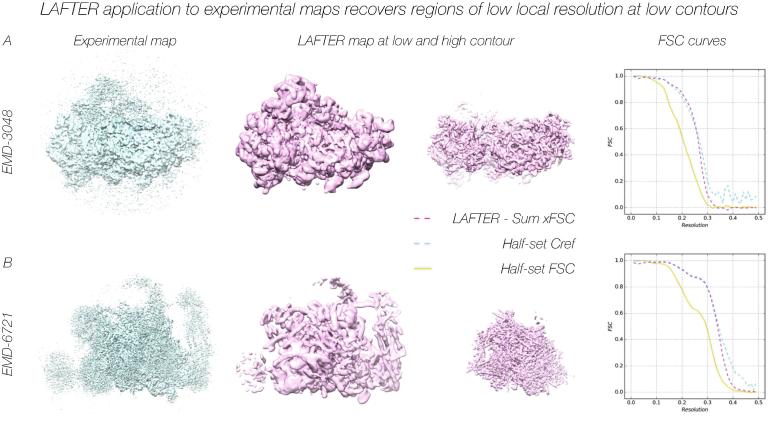
LAFTER application to experimental maps recovers regions of low local resolution at low contours. LAFTER filtered volumes for EMD-3048 (A) and EMD-6721 (B) respectively. In each case the density is shown as a surface representation, with the corresponding deposited density and a region of higher resolution density shown for the purposes of comparison. The experimental densities are highly disrupted given their low local resolution and required filtering or further processing to interpret in each case. Curves for the half-set FSC, C_ref_ and Filter-Sum xFSC are adjacent as described in the key.

**Fig. 5:**
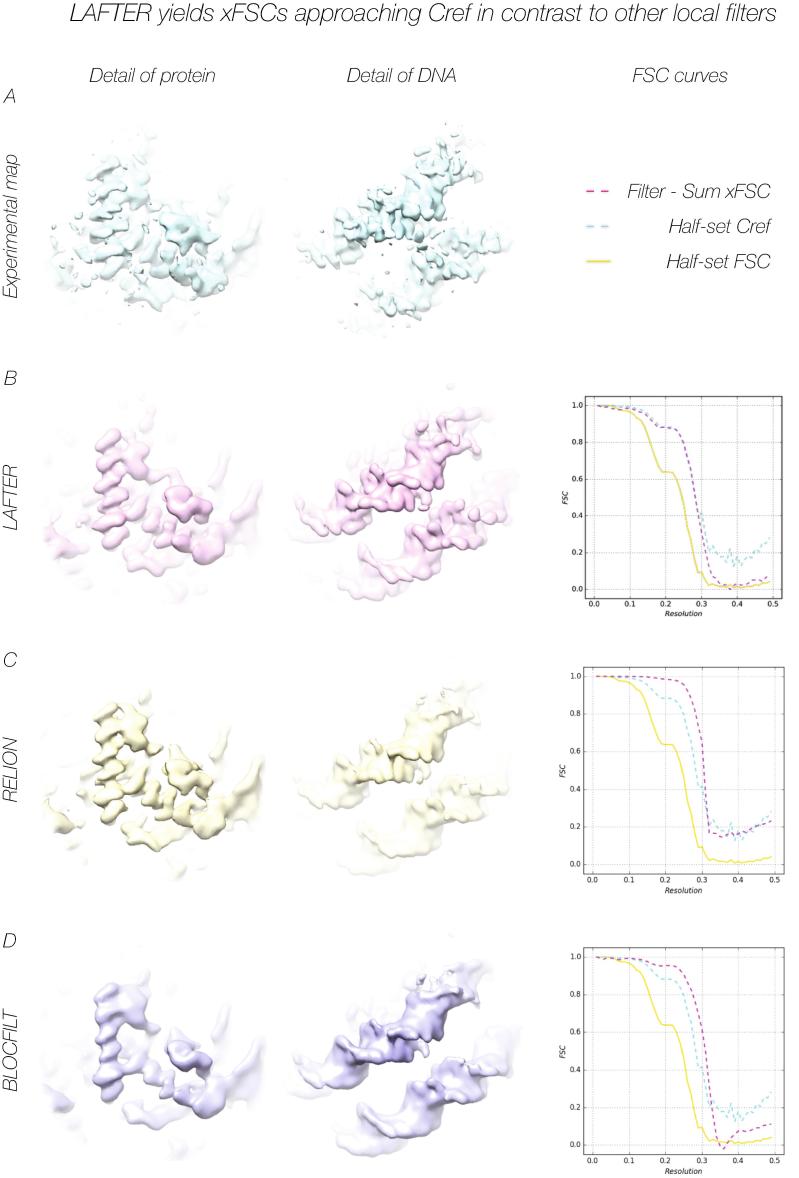
LAFTER yields xFSCs approaching Cref in contrast to other local filters. Regions of EMD-3954 representing protein structure and DNA structure are shown from globally filtered (A), LAFTER filtered (B), RELION local filtered (C) and BLOCFILT filtered (D) maps. One notable benefit of LAFTER is the recovery of stronger phosphate densities within the DNA backbone than are visible under the other local filtering approaches. Curves for the half-set FSC, C_ref_ and Filter-Sum xFSC in each case are adjacent and described in the key.

**Fig. 6:**
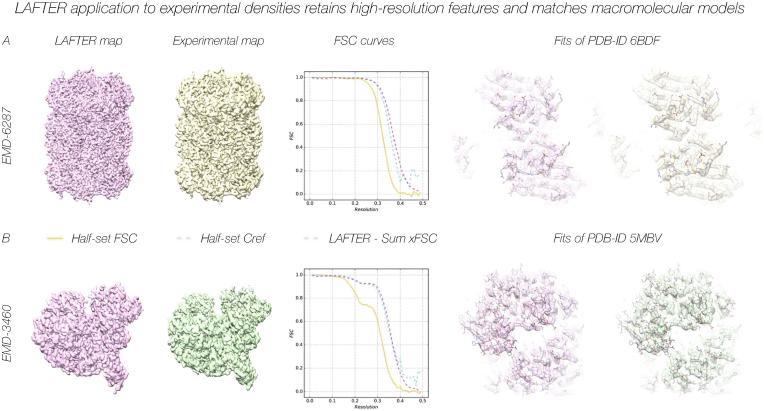
LAFTER application to experimental densities retains high-resolution features and matches macromolecular models. LAFTER filtered volumes and the original deposited maps are shown for EMD-6287 (A) and EMD-3460 (B). Detail of the corresponding models deposited in the PDB is shown fitted into the volumes in each case. Density is shown in a transparent surface representation with the atomic models (PDB-6BDF) and (PDB-5MBV) as skeletal models in CPK colours. Curves for the half-set FSC, C_ref_ and Filter-Sum xFSC in each case are adjacent as described in the key.

While for experimental data the noiseless volume against which C_ref_ is calculated is no more than a useful construct, it remains a useful parameter, since an FSC higher than C_ref_ indicates that noise is retained unsuppressed, whereas an FSC lower than C_ref_ indicates the loss of useful signal. The comparison between C_ref_ and the Filter-Sum xFSCs for five experimental datasets demonstrate a good match overall, the RMSD between the curves remaining consistently low with values of 0.018, 0.007, 0.006, 0.026 and 0.017 (Fig. 4A-B; Fig. 5B; Fig. 6A-B) respectively. Once again, although a systematic investigation is beyond the scope of this manuscript, this is not necessarily the case for other local filters, the RMSDs being 0.077 for RELION and 0.062 for BLOCFILT (Fig. 5C-D) respectively (see also Supp. Fig. 3B-C). Despite the good agreement shown by the low RMSD, inspection of the FSC curves for EMD-3048 (Fig. 4A) and EMD-6287 (Fig. 6A) reveals that the LAFTER-Sum xFSC curve is slightly above C_ref_ at resolutions below the C_ref_ = 0.5 threshold, indicating retention of more residual noise than would be preferred at these resolutions.

### 3.4 LAFTER reproducibly recovers low-SNR features from experimental datasets, and retains higher resolution features

The principal benefit of local filters such as LAFTER is the recovery of weak or low local-resolution features, which remain a substantial issue for macromolecular interpretation. To establish whether or not LAFTER fulfils this purpose, we trialled it on several datasets with regions of very low local resolution. LAFTER proved capable of robustly recovering low-resolution features from all reconstructions tested. Application of LAFTER to EMD-3048 (Llácer et al., 2015) in which the low-resolution features in the density map are resolved to 15–25 Å, whereas the high-resolution features extend to 4.9 Å (Fig. 4A), proved successful in recovering the envelope of peripheral factors bound to the 40S ribosomal subunit. Higher resolution features were retained well (Fig. 4A). While such extremely low-resolution density remains interpretable only as an envelope, higher-resolution rough or weak density can be rendered interpretable by filtering according to SNR. LAFTER output for EMD-6721 (Zhang et al., 2017), in which several peripheral sub-domains are resolved to 7–10 Å in comparison to the 3.6 Å overall resolution, filters these regions to retain secondary structural features (Fig. 4B). It should be noted that because LAFTER filters according SNR, lower local-resolution features appear at lower contours due to their weaker signal.

One of the notable benefits of the LAFTER approach is the recovery of high signal features in low SNR regions due to the fact that LAFTER is applied on a per-voxel basis explicitly terminated at the global resolution by filtering, whereas other “local-resolution” filters typically truncate the signal at the lowest resolution within a region. This is most noticeable in the recovery of phosphate densities from DNA or RNA such as those in EMD-3954 (Ayala et al., 2018) (Fig. 5). Such densities are frequently weak, as they do not exhibit the same extreme level of signal seen in X-ray crystallographic structures due to the different scattering properties of electrons, and when peripheral their signal can be much reduced by blending with the noisy solvent. LAFTER has proven successful in recovering weak phosphate densities in several cases.

### 3.5 LAFTER exhibits good high-resolution feature retention for experimental datasets and output densities match deposited models

It is extremely important that high-resolution features are also recovered, either entirely without, or with minimal, degradation, otherwise the output map will be of insufficient quality to be used for interpretation. The application of LAFTER to two very well resolved volumes, EMD-6287 (Campbell et al., 2015) and EMD-3460 (Wilkinson et al., 2016), demonstrated clean recovery of high-resolution features such as side-chain densities and main-chain carbonyls (Fig. 6A-B). We note that some slight feature degradation is visible in comparison to the final sharpened volumes used by the authors for interpretation in a few regions. In particular, very weak side-chain densities are sometimes suppressed in comparison to the sharpened final volume, whereas main-chain features appear typically to be recovered more strongly than before.

The fits of the LAFTER filtered maps for the experimental structures considered were compared to the fits of the deposited models (PDB-6BDF and PDB-5MBV) into each final density. In each case the models matched the LAFTER filtered density very well, and essentially all features represented in the models were apparent in the filtered output at some reasonable variation of the density threshold (Fig. 6A-B). Feature retention proved statistically comparable to that from other local filters with respect to the PDB as demonstrated by xFSCs with PDB-5MBV (Supp. Fig. 4A-C).

## 4 Discussion

### 4.1 LAFTER can aid the interpretation of experimental data exhibiting low SNR or variable local resolution

LAFTER typically functions sufficiently quickly and reproducibly for routine use, and suppresses disagreement between two independently refined half volumes robustly. Our results from idealised synthetic datasets demonstrate that the suppression of features in disagreement and the recovery of features in agreement is accomplished cleanly.

During testing, the only substantial issues with the use of LAFTER for local filtering have come from the explicit assumption that agreement and disagreement between half volumes represent distributions of the signal and noise respectively. This assumption can often be violated due to masking and symmetry artefacts, or any other sources of information transfer between half sets, introduced during the refinement process. These can result in the accumulation of correlated noise in independent half sets, which is falsely interpreted as signal. During an experimental structure determination the half set FSC and other statistics will already have been calculated at the point at which any local filter plays a role. We would therefore suggest that any observed over-refinement is best attended to by the experimenter, through modification of the refinement strategy to prevent this occurrence, not as an afterthought through a local filter. Other minor disadvantages of the algorithm include the fact that lower local resolution features appear at a much lower contour, although this represents a real phenomenon in terms of relative signal strength. The occasional retention of mask or filter waveforms from refinement is also apparent at extremely low contours. Furthermore, again at similarly low contours, specks of higher resolution noise can be retained in regions of very low local resolution at a rate proportional to the logarithm of the number of voxels in the map.

In order to ensure that our process is as robust as possible, the filtered density output by LAFTER is explicitly low-pass filtered at the end of the process to the resolution of the chosen FSC criterion, in order to truncate the signal at that point. LAFTER filtered volumes are clearly incompatible with atomic model refinement, given that the original signal and noise spectra are required for such purposes, and a warning is presented to users in the output of our implementation. LAFTER proved capable of recovering signal in agreement between half-sets despite considerable variation in signal, noise, and both the global and local resolution. There is a cost: a slight reduction in the highest resolution features. This must be expected given that noise suppression is the aim of the process. Features that lie within the noise distribution are suppressed by design, which is unavoidable if the retention of high levels of noise is not desired. Good agreement was attained up to resolutions close to the experimental cut-off, however, for all volumes examined.

Given that our intention is to aid the interpretation of weak density, a reference implementation of LAFTER has been made freely available. We believe that LAFTER will be beneficial for the cryo-EM community during the interpretation of density maps with weak features, substantial variations in local resolution and/or low SNR.

### 4.2 We propose that the fit of the Filter-Sum xFSC to C_ref_ represents a useful measure of effective noise suppression by a local filter

While several local filters are available, and more are understood to be under development, to the authors’ knowledge there are as yet no proposed criteria or metrics by which to judge their suppression of noise within the filtered output. We propose that the agreement between C_ref_ and the Filter-Sum xFSC provides a useful measure by which to judge the success of a local filter in achieving noise suppression in the output map.

While a xFSC value close to C_ref_ will almost certainly indicate some small loss of signal and some level of retention of residual noise, in the absence of a means of reliably de-convoluting the signal from the noise, we suggest that agreement between these curves is a sensible measure of the effectiveness of the noise suppression achieved by a local filter. We highlight, however, that because the signal and noise contributions are imperfectly distinguishable this parameter is not suitable as the target function for an optimisation algorithm. There is no unique solution, and it therefore has to be ensured that noise, rather than signal, suppression is being favoured by the filter. Noise suppression to this level should minimise the possibility of over-interpretation of the filtered volume.

We have shown that the xFSC with a noiseless synthetic volume is higher, after noise suppression to these levels using LAFTER, than that with the summed half maps, supporting the validity of our approach. Of course, with respect to real experimental data used for single particle analysis, the idealised, noiseless, volume hypothesised for C_ref_ cannot exist and is only useful as a construct. We take the correspondence between C_ref_ and the Filter-Sum xFSC, however, as an indication that the suppression of noise in disagreement has been achieved robustly using our algorithm. We would suggest that the C_ref_ to xFSC root mean squared deviation, and the corresponding curves, are imperfect but not unreasonable measures to be reported for local filtering in general as a measure of the level of noise suppression achieved, and therefore the level of care that should be taken in interpreting the output map.
