# Supplementary material for: A Local Agreement Filtering Algorithm for Transmission EM Reconstructions
Source: J Struct Biol. 2019 Jan 1;205(1):30–40. doi: 10.1016/j.jsb.2018.11.011 (PMC6351148; doi:10.1016/j.jsb.2018.11.011)
Supplement: Supplementary data 1 [file mmc1.pdf]

A Local Agreement Filtering Algorithm for Transmission EM Reconstructions

Supplementary Figures

Kailash Ramlaul[1], Colin M Palmer[2],
& Christopher H S Aylett[1]*

[1] Section for Structural Biology, Department of Medicine, Imperial College Road, South Kensington, London, SW7 2BB, United Kingdom

[2] Scientific Computing Department, Science and Technology Facilities Council, Research Complex at Harwell, Didcot, OX11 0FA, United Kingdom

* To whom correspondence may be addressed: c.aylett@imperial.ac.uk

Illustration of the effect of perfect global and local filters on the Filter-Sum xFSC

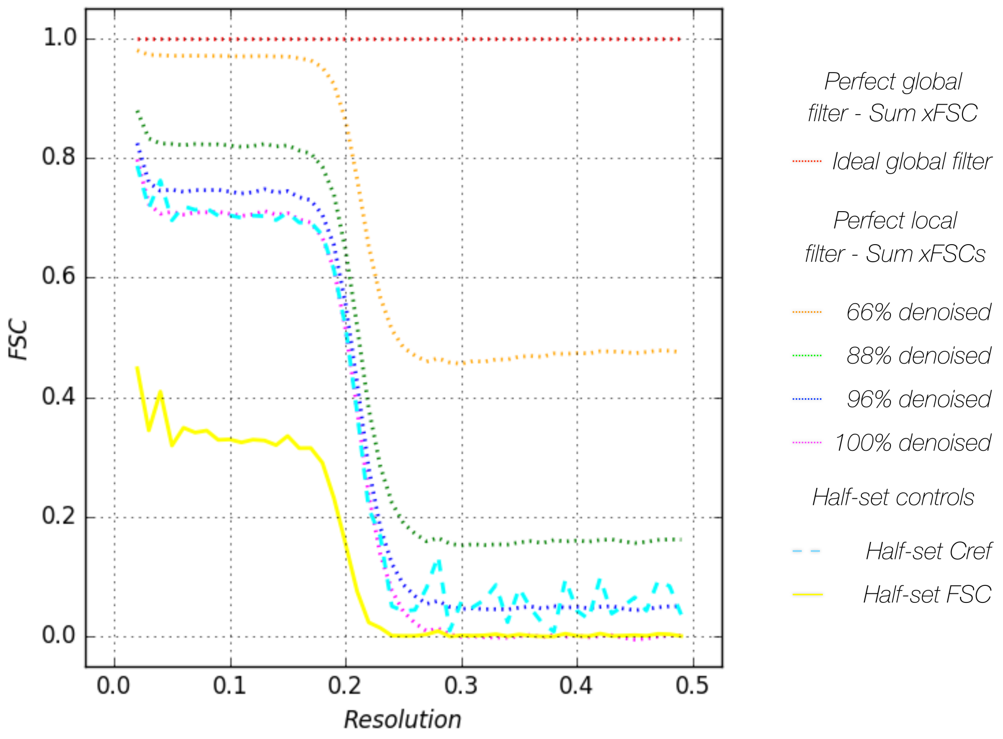

Supplementary Figure 1: Illustration of the effect of perfect global and local filters on the Filter-Sum xFSC. The noise within a map is progressively reduced and the resulting xFSC with the original noisy volume shown in each case. The half-set FSC and C_{ref} are also shown to highlight the relationship between C_{ref} and the xFSC for a volume within which noise has been effectively suppressed.

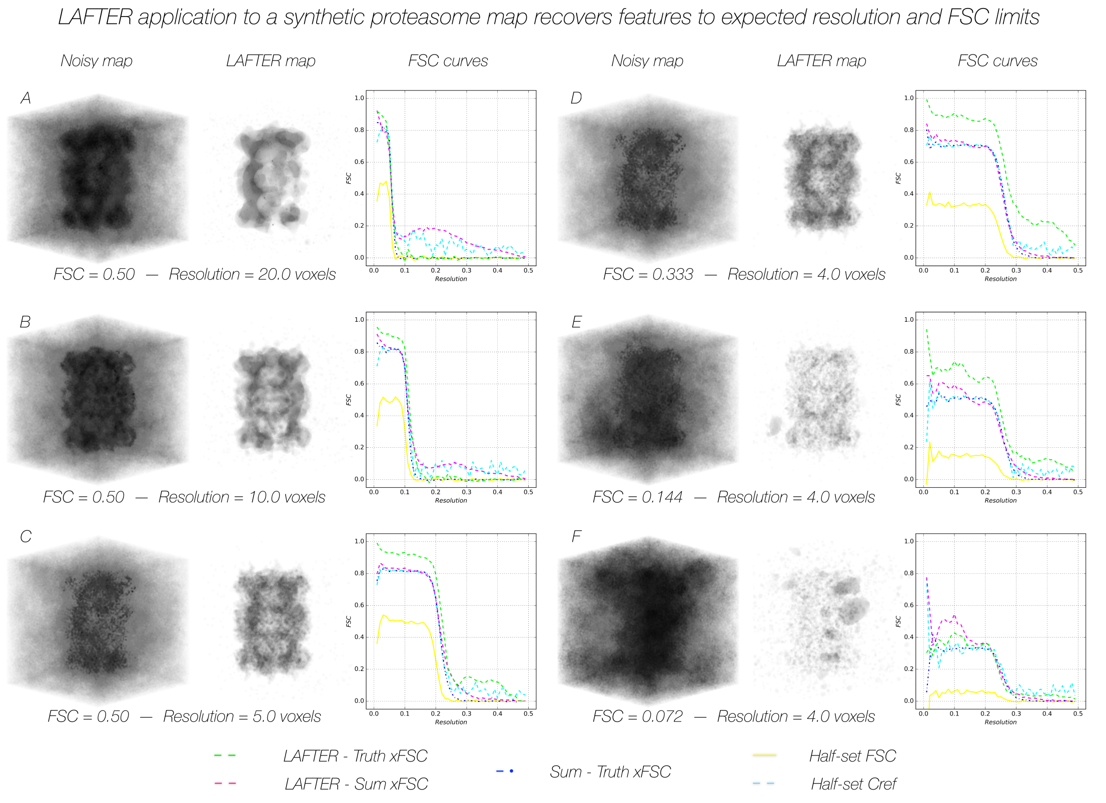

Supplementary Figure 2: LAFTER application to a synthetic proteasome map recovers features to expected resolution and FSC limits. To demonstrate that the type of synthetic density is unimportant, figures 2 and 3 are recapitulated with a synthetic proteasome. LAFTER output for synthetic input truncated at 20 voxels (A), 10 voxels (B) and 5 voxels (C) and with FSCs of 0.333 (D), 0.144 (E) and 0.072 (F). In each case the noisy volume and the filtered density are shown as transparent “solids” as the signal in the half volumes is weak. The half-set FSC, C_{ref} and xFSCs between the filtered, true and summed densities in each case are shown adjacent as described in the key.

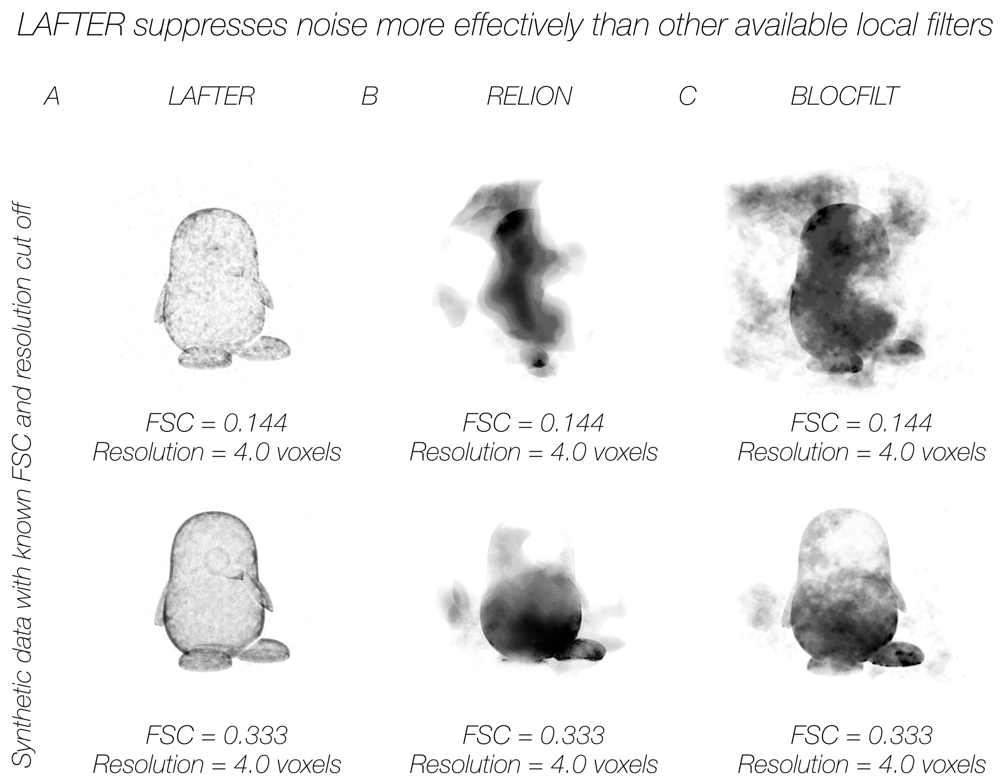

Supplementary Figure 3: LAFTER suppresses noise more effectively than other available local filters. The output of the RELION (B) and BLOCFILT (C) local filters for the FSC 0.144 and 0.333 synthetic densities is compared to that of LAFTER (A).

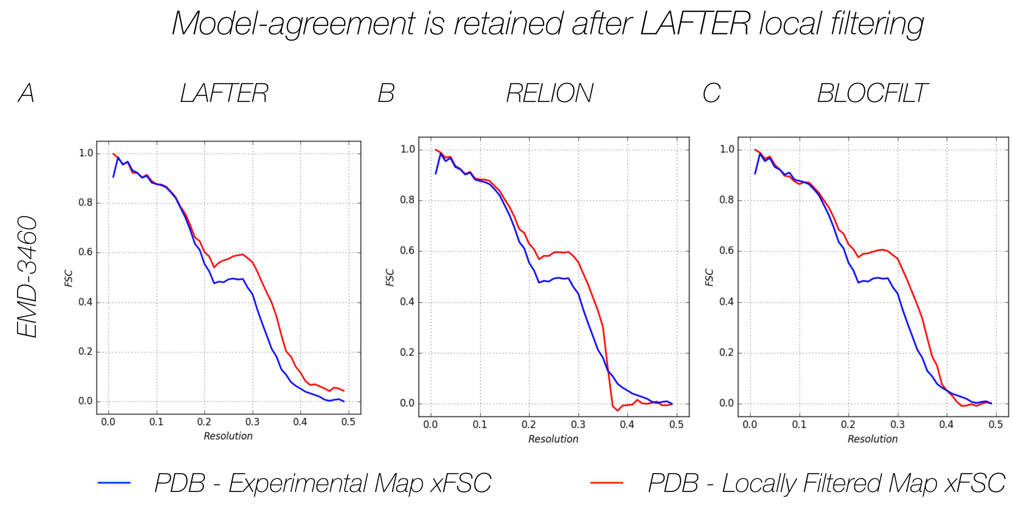

Supplementary Figure 4: Model-agreement is retained after LAFTER filtering. xFSC curves calculated between the indicated filtered density and the theoretical protein density of PDB-5MBV for LAFTER (A), RELION (B) and BLOCFILT (C). The filtered-volume to PDB-volume xFSC is shown in red in each case, while the xFSC with the unfiltered density is shown in blue for comparison.

Acknowledgments

The authors would like to thank; R. Ayala, R. Glyde, M. Wilkinson, D. Wigley and X. Zhang for providing maps for beta testing of LAFTER on problematic electron microscopy datasets, thingiverse users aeron203, luigismith and me2space for the noiseless volumes used for synthetic data, D. Boehringer, L. Frankland, B. Greber, M. Itten, M. A. Leibundgut, E. Morris and A. Scaiola for their helpful commentary, identification and discussion of flaws preventing adoption of our previous approaches to facilitate the interpretation of noisy densities, and M. Winn for proof-reading the manuscript.

Funding

This work was funded by the Wellcome Trust and the Royal Society through a Sir Henry Dale Fellowship (206212/Z/17/Z) to CHSA. CMP is supported by Medical Research Council funding (MR/N009614/1).

Conflict of interest statement

The authors declare that they know of no conflicts of interest with respect to this work.
